# Critical role of zinc finger protein 521 in the control of growth, clonogenicity and tumorigenic potential of medulloblastoma cells

**DOI:** 10.18632/oncotarget.1176

**Published:** 2013-07-27

**Authors:** Raffaella Spina, Gessica Filocamo, Enrico Iaccino, Stefania Scicchitano, Michela Lupia, Emanuela Chiarella, Tiziana Mega, Francesca Bernaudo, Daniela Pelaggi, Maria Mesuraca, Simonetta Pazzaglia, Samantha Semenkow, Eli E. Bar, Marcel Kool, Stefan Pfister, Heather M. Bond, Charles G. Eberhart, Christian Steinkühler, Giovanni Morrone

**Affiliations:** ^1^ Laboratory of Molecular Haematopoiesis and Stem Cell Biology, Dept. of Experimental and Clinical Medicine, University of Catanzaro Magna Græcia, Catanzaro, Italy.; ^2^ Exiris srl, Via Castelfidardo 8, Rome, Italy.; ^3^ Department of Pathology, Johns Hopkins University School of Medicine, Baltimore MD, USA.; ^4^ Section of Toxicology and Biomedical Sciences, ENEA CR-Casaccia, Via Anguillarese 301, S. Maria di Galeria (Roma), Italy.; ^5^ Division of Pediatric Neurooncology, German Cancer Research Center, Im Neuenheimer Feld 280, Heidelberg, Germany.; ^6^ Department of Pediatric Oncology, Hematology and Immunology, University Hospital Heidelberg, Heidelberg, Germany; ^7^ Current address: Department of Neurological Surgery, Case Western Reserve University, Cleveland, Ohio, USA.

**Keywords:** ZNF521, gene expression, medulloblastoma, cell growth, tumorigenicity, cancer stem cells

## Abstract

The stem cell-associated transcription co-factor ZNF521 has been implicated in the control of hematopoietic, osteo-adipogenic and neural progenitor cells. *ZNF521* is highly expressed in cerebellum and in particular in the neonatal external granule layer that contains candidate medulloblastoma cells-of-origin, and in the majority of human medulloblastomas. Here we have explored its involvement in the control of human and murine medulloblastoma cells.

The effect of ZNF521 on growth and tumorigenic potential of human medulloblastoma cell lines as well as primary *Ptc1*^−/+^ mouse medulloblastoma cells was investigated in a variety of *in vitro* and *in vivo* assays, by modulating its expression using lentiviral vectors carrying the ZNF521 cDNA, or shRNAs that silence its expression.

Enforced overexpression of *ZNF521* in DAOY medulloblastoma cells significantly increased their proliferation, growth as spheroids and ability to generate clones in single-cell cultures and semisolid media, and enhanced their migratory ability in wound-healing assays. Importantly, ZNF521-expressing cells displayed a greatly enhanced tumorigenic potential in nude mice. All these activities required the ZNF521 N-terminal motif that recruits the nucleosome remodeling and histone deacetylase complex, which might therefore represent an appealing therapeutic target. Conversely, silencing of *ZNF521* in human UW228 medulloblastoma cells that display high baseline expression decreased their proliferation, clonogenicity, sphere formation and wound-healing ability. Similarly, *Zfp521* silencing in mouse *Ptc1*^−/+^ medulloblastoma cells drastically reduced their growth and tumorigenic potential.

Our data strongly support the notion that ZNF521, through the recruitment of the NuRD complex, contributes to the clonogenic growth, migration and tumorigenicity of medulloblastoma cells.

## INTRODUCTION

Zinc finger protein 521 (EHZF/ZNF521) is a transcription co-factor originally identified for its abundant and selective expression in early progenitors of the human hematopoietic system [1]. ZNF521 contains 30 zinc fingers and an N-terminal motif of 12 amino acids that this factor shares with numerous transcriptional co-repressors [1, 2] most of which are endowed with a recognized regulatory role in diverse developmental processes. This motif recruits the nucleosome remodeling and histone deacetylase (NuRD) complex [3, 4] and is required for the co-repressor function of Friend of GATA (FOG)-1 [5, 6], SALL1 [7] and SALL4 [8].

In the hematopoietic system ZNF521 is highly expressed in stem and progenitor cells but not in more differentiated precursors or mature leukocytes [1, 4]. Its roles thus far documented in this system include the inhibition of erythroid differentiation mediated by the repression of GATA1 target genes [9] and the inhibition of the transcriptional activity of early B-cell factor 1 (EBF1) accompanied by the modulation of the B-lineage maturation of primitive hemo-lymphopoietic progenitors [10]. In mice, Zfp521 has been implicated in the development of B-lymphoid malignancies including B-cell lymphomas [11] and acute B-cell leukemias – the latter in co-operation with the *E2A-HLF* fusion oncogene [12].

A growing body of evidence indicates that Zfp521 is a central lineage choice determinant in mesenchymal stem cells, where, through a complex network of physical and functional interactions with Zfp423, Ebf1 and Runx2, combined with the contribution of a variety of chromatin remodeling factors, it promotes osteogenesis at the expense of adipose differentiation [13-17].

High abundance of zinc finger protein 521 is observed in brain [1] and in neural stem cells [4] as well as in striatonigral neurons [18]. In the precursors of these cells, *Zfp521* expression parallels that of *Ebf1* which is essential for their differentiation, suggesting that the interplay of the two factors may control the homeostasis of the immature striatal compartment. Additional evidence delineating a central role for Zfp521 in neurogenesis has come from a recent study where Kamiya et al. [19] demonstrated that this factor dictates the spontaneous generation of neuroectodermal precursors from embryonic stem cells, and that its silencing abrogates their neural potential.

Among all brain regions, the highest expression of *ZNF521/Zfp521* is observed in cerebellum. During postnatal cerebellar development in mouse, the *Zfp521* transcript is highly enriched in the external granule layer that hosts the cerebellar granule neuron precursors [4]. Intriguingly, disruption of the gene encoding the *Zfp521* paralogue (*Zfp423/Oaz*) is associated with severe cerebellar defects in mice [20-22] suggesting that both factors may participate with non-redundant functions in the control of development in this organ.

Cerebellar granule neuron precursors are believed to represent the cells-of-origin for a substantial fraction of medulloblastomas (MBs), the most frequent malignant brain tumor of childhood. Given the high expression of *ZNF521/Zfp521* in the cerebellum - and especially in the external granule layer during its development [4] - we decided to investigate the role of ZNF521 in medulloblastoma. The results of this study highlight a strong relationship between activity of this factor and growth and tumorigenic potential of human and mouse medulloblastoma cells, indicating that ZNF521 is likely to play a role in the pathogenesis of this tumor.

## RESULTS

### Abundant expression of *ZNF521* in human medulloblastomas

**Figure 1:**
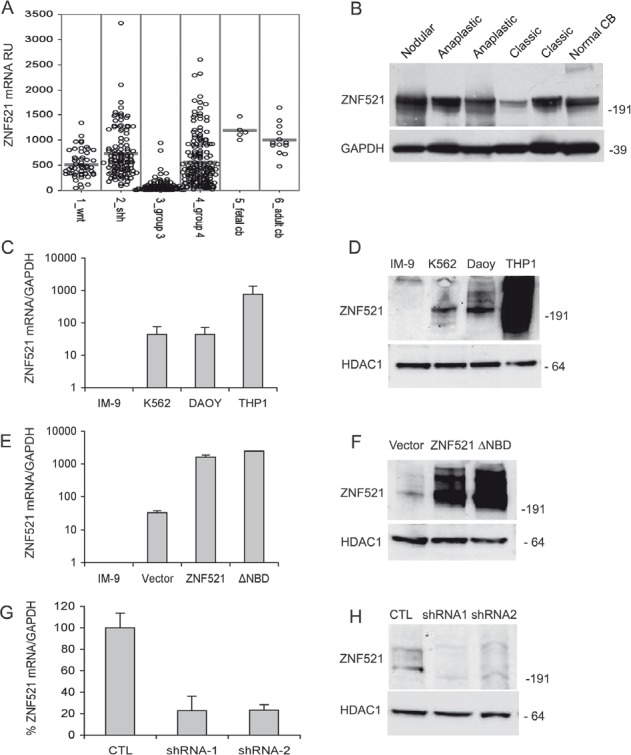
Expression of ZNF521 in human medulloblastomas and modulation of its expression of in DAOY medulloblastoma cells Panel A: expression of ZNF521 mRNA in subsets of human medulloblastomas and in human fetal and adult cerebellum. The data were analyzed as described in materials and methods. Panel B: expression of ZNF521 protein in human medulloblastomas and in non-neoplastic cerebellum. Western blotting analysis was performed using the S15-EHZF antibody. This experiment was conducted on total cell extracts and GAPDH was used as an internal control. Panels C and D: the endogenous expression of ZNF521 in DAOY cells was compared by Q-RT-PCR (C) and western blotting (D) with that of the B-lymphoblastoid cell line IM-9 that produces extremely low levels of both mRNA and proteins, the erythro-myeloid K-562 cells that display moderate expression, and the myelo-monocytic THP1 cells that express high levels of ZNF521. In (D) the endogenous ZNF521 protein was detected using the S15-EHZF antibody. Panels E and F: the expression of ZNF521 was measured by Q-RT-PCR (E) and western blotting (F) in DAOY cells transduced with void FUIGW vector (referred to in all figures as Vector), FUIGW-ZNF521 (ZNF521) and FUIGW- ZNF521ΔNBD (ΔNBD). In (D) the transduced protein was detected using an S15 anti-EHZF antibody. Panels G and H: the expression of ZNF521 was measured by Q-RT-PCR (G) and western blotting (H) in DAOY cells transduced with the vector FG12 (CTL) and the shRNA-containing FG12-H11 (shRNA1) and LV-H85 (shRNA2). In (H) the endogenous ZNF521 protein was detected using the S15-EHZF antibody. In all western blotting experiments shown HDAC1 was used as an internal control. The data shown here illustrate a representative experiment of sets of at least 3.

Numerous recent studies have delineated the existence of distinct molecular subgroups of medulloblastomas based on their specific gene expression profiles [23-31]. We examined the *ZNF521* mRNA expression levels in a series of previously published analyses [23-26] as well as unpublished data, comprising a total of 436 cases of medulloblastomas and 18 normal cerebellum specimens. The results (Fig 1, panel A) show that, with the exception of Group 3 MBs, *ZNF521* expression in medulloblastomas is comparable to that of adult cerebellum, with a considerable fraction of the tumors in the SHH subgroup and Group 4 MBs displaying high expression levels. Western blotting analysis of 5 medulloblastoma specimens in comparison with non-neoplastic cerebellar tissue confirmed the presence of equivalent amounts of ZNF521 protein (Fig 1 B).

### Modulation of ZNF521 activity in DAOY human medulloblastoma cells regulates growth in adherent- and anchorage-independent culture conditions

To investigate the role of ZNF521 in the regulation of human MB cells we used the DAOY cell line, which was derived from a biopsy of desmoplastic medulloblastoma [32]. We first compared, by quantitative RT-PCR and Western blotting, the levels of *ZNF521* mRNA and protein in DAOY with those of the leukemic cell lines IM-9, K562 and THP1, that express low/undetectable, intermediate and high amounts of this factor, respectively. This analysis (Fig. 1, panels C and D) revealed moderate *ZNF521* expression in DAOY cells, comparable to that of K562 and considerably lower than that of THP1.

In gain of functions studies, DAOY cells overexpressing ZNF521 or controls with mutant protein unable to recruit the NuRD complex were examined. Loss of function was assessed after ZNF521 expression was silenced by RNAi. When DAOY cells were transduced with lentiviral vectors carrying the cDNAs encoding ZNF521 or its deletion mutant lacking the NuRD-binding motif (ZNF521ΔNBD), an over 25-fold increase in the abundance of the relevant mRNA and protein was detected (Fig. 1, E and F). Conversely, transduction of DAOY with lentiviruses containing two distinct ZNF521 shRNAs (H11 and H85) reduced mRNA expression by >70% and strongly decreased the expression of the protein (Fig. 1, G and H).

**Figure 2:**
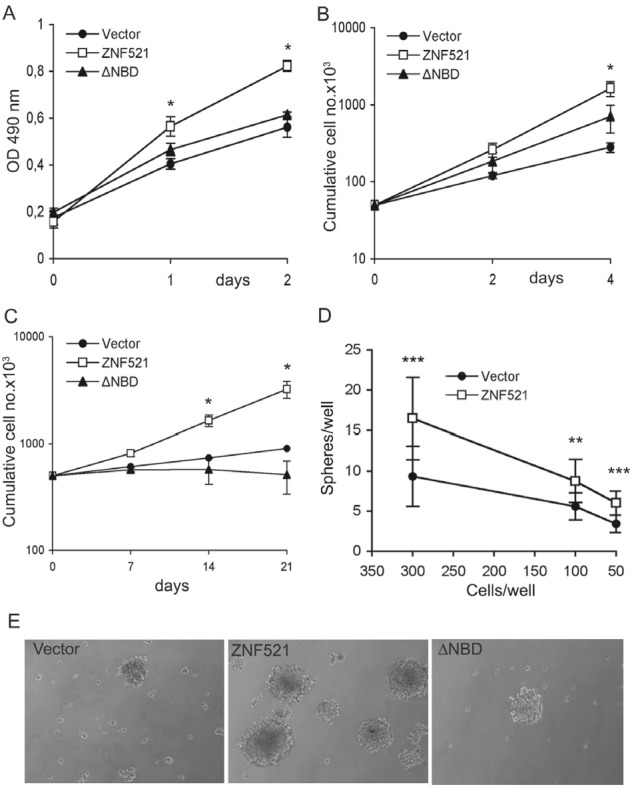
Enforced expression of ZNF521 confers growth advantage on DAOY cells Panels A and B: growth of DAOY cells transduced with FUIGW, FUIGW-ZNF521 and FUIGW-ZNF521ΔNBD cultured in adherent conditions. Growth was assessed by MTS assays (A) or by cell counts (B) as described in the materials and methods. Panel C: Growth of DAOY cells transduced with FUIGW, FUIGW-ZNF521 and FUIGW- ZNF521ΔNBD in anchorage-independent conditions. The cells were propagated in neurosphere-like cultures as described in materials and methods. At the indicated times the cells were carefully resuspended in single-cell suspensions, counted and replated at equal densities. Panel D: DAOY cells FUIGW or FUIGW-ZNF521 were plated at stepwise dilutions (12 replicates per dilution) in low-adherence 48-well plates, starting at 3,000/well. The cultures were examined twice per week, and fresh complete medium was added. Spheres were enumerated after 15 days of culture. Cell concentrations above 300/well gave rise to an excessive number of spheres, that could not be counted. Panel E: representative spheroids from the three cultures of transduced DAOY cells. The data shown here illustrate a representative experiments of sets of 6 (Panel A), 3 (B) and 3 (C). *: p<0.05 **: p<0.005; ***: p<0.0005

We first measured growth in adherent cultures using MTS assays and cell counts, and found that overexpression of ZNF521 induced a significant increase in the growth rate (Fig. 2 panels A and B) whereas the ΔNBD mutant elicited a minor and non significant increase. A more pronounced difference was induced by ZNF521 but not by ZNF521ΔNBD when the cells were grown as spheroids in anchorage-independent conditions. In these assays the cultures overexpressing ZNF521 contained a higher total numbers of cells (Fig. 2C) and of spheres - and larger spheres (Fig. 2E) - than the control cultures or the ZNF521ΔNBD-expressing cells. It is interesting to notice that in this assay the growth of DAOY transduced with ZNF521ΔNBD was comparable or even lower than that of the cells transduced with the control virus, resulting in the extinction of the cultures after a limited number of passages (not shown). When the sphere formation assay was performed in several limiting dilution conditions, we observed a consistent, 50-80% increase in the average number of spheres (Fig. 2D) and a larger sphere size (not shown) in the ZNF521-transduced cultures compared to the controls.

### ZNF521 enhances clonogenicity of DAOY medulloblastoma cells.

**Figure 3:**
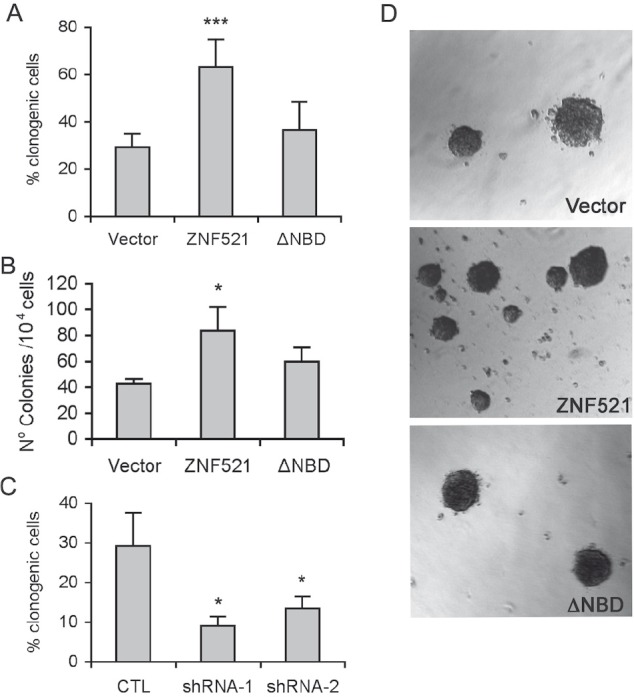
ZNF521 enhances clonogenicity of DAOY cells Panel A: clonogenicity of DAOY cells transduced with FUIGW, FUIGW-ZNF521 and FUIGW- ZNF521ΔNBD in single-cell culture conditions. Cells were plated in 96-well plates at an average density of 0.33 cells/well. Clones containing ≥100 cells were scored two weeks after plating. Panel B: clonogenicity of DAOY cells transduced with FUIGW, FUIGW-ZNF521 and FUIGW- ZNF521ΔNBD in soft agar. 10^4^ cells/plate were seeded. After 3–4 weeks, colonies larger than 50 μM were scored as described in materials and methods. Panel C: clonogenicity of DAOY cells transduced with FG12, FG12-H11 (shRNA1) or LV-H85 (shRNA2) in single-cell culture conditions. Panel D: representative colonies obtained in soft agar assays. The data shown here illustrate representative experiments of a set of 15 (panel A), 3 (panel B) and 6 (panel C). *: p<0.05; ***: p<0.0001

Sphere-forming ability does not always represent an accurate and reliable index of a cell's clonogenic potential, as spheres are prone to fragmentation or fusion [33-34]. Therefore, to quantitatively assess the effect of ZNF521 on the clonogenicity of DAOY cells we measured the capacity of these cells to generate clones at the single-cell level by: i) single-cell adherent cultures, and ii) anchorage-independent clonogenic assays in soft agar. As illustrated in Fig. 3 (panel A: single-cell adherent clones, panel B and D: soft agar clones), both assays revealed a significant, over two-fold increase in the frequency of clonogenic cells among those overexpressing ZNF521 compared to the vector control and ZNF521ΔNBD-expressing cells. Conversely, silencing of *ZNF521* resulted in a 2 to 3-fold decrease in DAOY clonogenicity in limiting dilution assays (Fig. 3C).

### ZNF521 stimulates migration of DAOY medulloblastoma cells.

**Figure 4:**
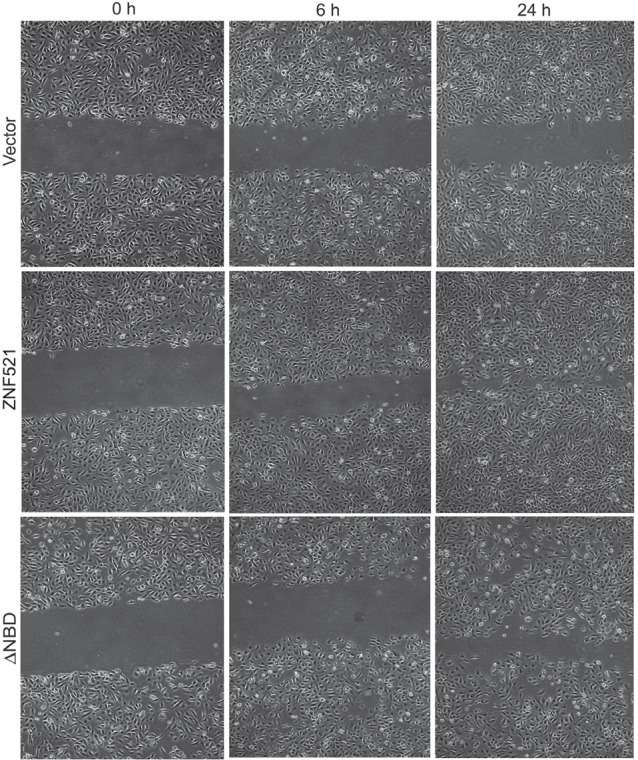
ZNF521 enhances the migratory potential of DAOY cells The wound-healing assays were performed as described in materials and methods. This figure illustrates a representative experiment of a set of 5.

The effect of ZNF521 on the migratory potential of DAOY cells was investigated using wound-healing assays. To this end, a scratch was produced in confluent monolayers of cells transduced with control vector, ZNF521 or ZNF521ΔNBD, and the cultures were observed at regular time intervals to monitor the rate of “healing”. As shown in Fig. 4, DAOY expressing ZNF521 displayed a much higher migration across the scratch, whose width was reduced by approximately 50% within 6 hours and almost completely covered by 24h. In contrast, the scratch was still evident after 24 hours in cultures transduced with ZNF521ΔNBD and those infected with void vector. To rule out the possibility that this effect could be due to the higher proliferation rate of ZNF521-transduced cells, the experiment was repeated in the presence of mitomycin C. As documented in Supplementary Fig. 1, in these conditions ZNF521-expressing DAOY cells also exhibited a higher wound-healing capacity than those transduced with control vector.

### Effect of ZNF521 on growth, clonogenicity and migration of additional human medulloblastoma cell lines

To further confirm the results obtained in the studies illustrated above we used UW228 cells, also widely employed as models of human medulloblastoma. Since these cells displayed considerably higher baseline expression of *ZNF521* than DAOY cells (Supplementary Fig. 2, panel A) we knocked down *ZNF521* expression with the shRNAs H11 (shRNA1) and H85 (shRNA2) (Sup. Fig. 2B). Both shRNAs caused a significant decrease in UW228 growth (Sup. Fig. 2C), clonogenicity in single-cell conditions (Sup. Fig. 2D) growth as spheroid (Sup. Fig. 2, E and F) and wound-healing capacity (Sup. Fig. 2G). To extend our gain of function studies to an additional human cell line we used D283 MB cells, which have low baseline expression of *ZNF521*. Consistent with the notion that this stem cell-associated factor promotes medulloblastoma growth, enforced expression of ZNF521 in D283 cells (Sup. Fig. 3A) resulted in a consistent increase in their proliferation (Sup. Fig 3B).

### ZNF521 strongly enhances *in vivo* tumorigenicity of DAOY medulloblastoma cells

**Figure 5:**
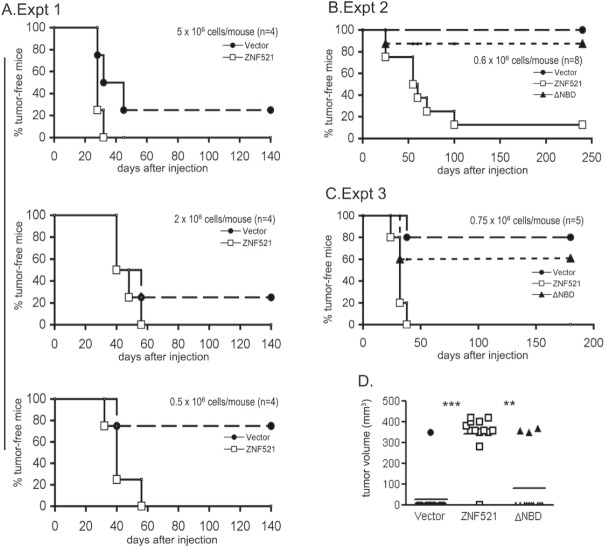
ZNF521 enhances the tumorigenicity of DAOY cells in nude mice Xenografts of DAOY transduced as indicated were performed and monitored as described in materials and methods. The graphs (A, B and C) illustrate the tumor-free intervals for the three experiments described in the Results. The symbols indicate: solid circles: control vector (FUIGW); open squares: FUIGW-ZNF521; solid triangles: FUIGW-ZNF521ΔNBD. Panel D illustrates the aggregate data from exp. 2 and 3. Tumor volume at the time of sacrifice is indicated. P values of tumor vs. no tumor development in these experiments were: P = 3.269 × 10^−5^ for Vector vs. ZNF521, and P = 0.00098 for ZNF521ΔNBD vs. ZNF521.

The data thus far presented demonstrate that ZNF521 is a relevant regulatory factor in DAOY: its enforced expression confers a strong proliferative advantage on these cells that is mirrored by their increased ability to generate clones both in adherent conditions and in soft agar as well as spheroids, and by enhanced wound-healing ability. In all these activities the integrity of the NuRD-binding motif located in the amino-terminal portion of ZNF521 appears to be essential. To test if the overexpression of *ZNF521* is also associated with increased *in vivo* tumorigenicity in DAOY cells, we carried out subcutaneous xenograft studies in nude mice. The initial experiment was performed using different amounts of cells transduced with FUIGW vector or with FUIGW-ZNF521 (5×10^5^, 2×10^6^ and 5×10^6^ cells/mouse respectively), to establish which cell dosage would be unable to generate tumors in 100% of the recipient mice, and therefore provide an optimal condition to highlight any differences in tumorigenic potential between the cell populations injected. In this experiment, tumors were observed in all animals inoculated with ZNF521-DAOY irrespective of the number of cells. In contrast, 3/4 mice in each cohort inoculated with high doses (5×10^6^ and 2×10^6^) of FUIGW-DAOY, and only 1/4 in the group injected with 5×10^5^ cells, developed detectable tumors (Fig. 5A).

To confirm and extend these data, in further experiments larger cohorts of 8 and 5 mice were inoculated with 6×10^5^ and 7.5×10^5^ cells per mouse respectively. In these assays, DAOY expressing ZNF521ΔNBD were also included. As shown in Fig. 5 (panels B and C), the cells expressing wild-type ZNF521 demonstrated in both experiments a much higher tumorigenic potential than those transduced with the ΔNBD mutant, which were only slightly superior to the control cells in their ability to generate tumors in nude mice. The aggregate data of Exp. 2 and Exp. 3, summarized in panel D of Fig. 5, demonstrate a highly significant difference in tumorigenicity between DAOY cells transduced with ZNF521 and cells infected with control vector or ZNF521ΔNBD. All tumors analyzed displayed similar histology (Sup. Fig. 4A), and were cellular lesions comprised of poorly differentiated cells with limited cytoplasm. Immunohistochemical analyses with anti-ZNF521 antibody demonstrated the presence of high levels of the protein in virtually all cells in the tumors deriving from DAOY-ZNF521 and DAOY-ZNF521ΔNBD compared to those generated by control cells (Sup. Fig. 4B) expressing only the endogenous protein.

### Zfp521 silencing impairs growth and tumorigenicity of Ptc^+/−^ mouse medulloblastoma cells

A wealth of mouse models have been generated to study the biology of brain tumors [35] and in particular of medulloblastomas [36]. To gain further evidence of the involvement of ZNF521 in the pathogenesis of medulloblastoma, we exploited the availability of one of these models and investigated the role of Zfp521 in primary mouse MB cells. Mice with haploinsufficiency of the *Ptc gene* that encodes the regulatory subunit of the hedgehog (HH) receptor complex are prone to the development of cerebellar tumors that recapitulate the features of human medulloblastoma [37-39]. Cells derived from these tumors, if injected subcutaneously, give rise to neoplasms that retain the features of the parental cancer [40]. We have found that these cells can also be propagated ex vivo for brief periods of time using neurosphere culture conditions, without losing their sensitivity to HH inhibitors nor their tumorigenic potential (Filocamo et al., unpublished data). Loss of HH pathway dependence is usually observed in human medulloblastoma cell lines. Therefore, the intactness of the HH signaling pathway in our short term *Ptc*^+/−^ MB serum-free cultures makes these cells a useful model of HH-dependent medulloblastomas.

**Figure 6:**
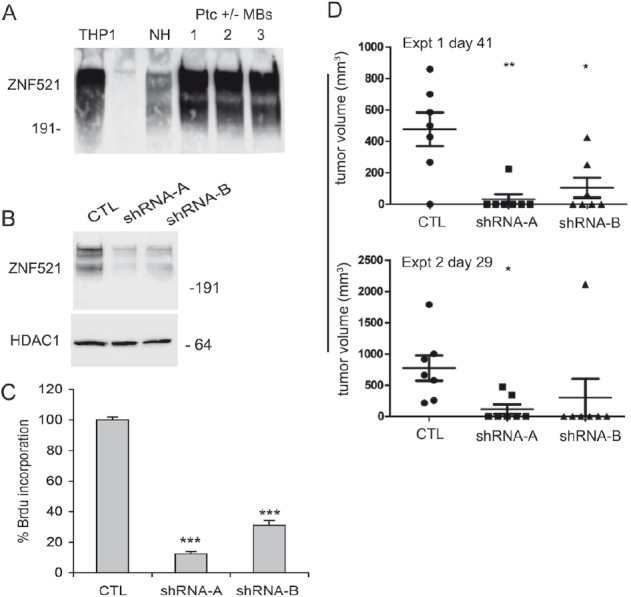
Silencing of Zfp521 impairs growth and tumorigenicity of Ptc^−/+^ mouse medulloblastoma cells Panel A: the expression of Zfp521 in three distinct extracts of Ptc^−/+^ medulloblastoma cells was assessed by western blotting using the I88 antibody as detailed in the materials and methods. As controls, human THP1 and mouse NIH 3T3 cells were used. Panel B: western blotting analysis of Zfp521 expression in transduced with lentiviral vectors carrying a control no target shRNA (CTL) or two specific Zfp521 shRNAs (LVP15 and LVP19). For the detection of Zfp521, the I88 antibody was used. Panel C: BrdU incorporation in Ptc^−/+^ medulloblastoma cells transduced with lentiviral vectors carrying a control no target shRNA (CTL) or two specific Zfp521 shRNAs (LVP15 and LVP19, referred to as shRNA-A and shRNA-B respectively) was assayed as detailed in materials and methods. Results from a representative experiment of a series of five are shown; both LVP15 (shRNA–A) and LVP19 (shRNA–B) are significantly different from CTL, with a p-value < 0.0001 (***). Panel D: tumor growth in CD1 nude mice injected each with 5×10^4^ Ptc^−/+^ medulloblastoma cells transduced with lentiviral vectors carrying either a control no target shRNA (CTL) or two specific Zfp521 shRNAs (LVP15 and LVP19: shRNA A and B respectively). The figure illustrates two independent experiments on cohorts of seven mice each. The injections and tumor monitoring are detailed in materials and methods.

The expression levels of Zfp521 in Ptc^+/−^ MBs were assayed by Western blotting analysis using an antibody to an internal ZNF521 peptide that recognizes both the murine and human proteins in their native non-reduced form [10]. Abundant expression was detected in all three tumor samples tested, comparable to that displayed by the human THP1 and murine NIH 3T3 cells used as positive controls (Fig. 6A). This seems consistent with the high levels of *ZNF521* expression observed in the subgroup 2 of human MBs, that is characterised by dysregulation of the SHH pathway (Fig. 1A).

For *Zfp521* knock-down, MB cells isolated from the tumors of *Ptc1*^+/−^ mice were infected with lentiviral vectors (LVP15 and LVP19) carrying the sequences for two *Zfp521*-specific shRNAs or with a control lentivirus containing a non-target shRNA. Fig 6B illustrates the strong reduction of the Zfp521 levels in cells transduced with both *Zfp521*-specific shRNAs in comparison to those transduced with the control vector.

The transduced cells were cultured in anchorage-independent conditions and their growth was assessed by measuring the incorporation of 5-bromo-2'-deoxyuridine. As documented in Fig. 6C, the proliferation rate of cells where *Zfp521* expression had been silenced was significantly reduced (>60-80% compared to the controls), indicating a role for Zfp521 in the regulation of the growth of these cells.

We then asked whether Zfp521 was required for (or contributed to) the tumorigenicity of *Ptc1*^+/−^ MB cells. To this end, 5×10^4^ cells transduced with the shRNAs described were injected subcutaneously in CD1 nude mice. Tumor growth was monitored at regular intervals. The results of two distinct experiments on cohorts of seven mice each, illustrated in Fig. 6D, demonstrate a considerable reduction in tumor initiation and average size in the mice injected with *Zfp521*-silenced cells compared to the controls.

## DISCUSSION

Medulloblastoma is a composite group of malignant brain tumors of embryonal origin with heterogeneous clinical and molecular features. A variety of dysregulated mechanisms are believed to contribute to its pathogenesis and to the homeostasis of the malignant cell population [41-44]; the existence of multiple major pathogenetic mechanisms is mirrored by the existence of distinct MB subgroups characterised by specific gene expression profiles [23-31, 45]. Recent experimental evidence implies that different subgroups may arise from transformation of specific types of neural progenitors [46]. In the light of its abundance in immature neural cells (including cerebellar granule neurons precursors [4]) and in the majority of human medulloblastomas (Fig. 1 A and B), and of its recognized role as one of the earliest inducers of neural development [19], ZNF521 could be a factor involved in this process. To address this possibility we investigated the effects of ZNF521 in DAOY, a cell line commonly employed as a model for human medulloblastoma whose moderate expression of ZNF521 makes it amenable to the study of the biological effects of both overexpression and knock-down of this factor. We subsequently validated the gain and loss of function results of these studies in D283 and UW228 lines, respectively.

Overexpression of *ZNF521* significantly enhanced the DAOY growth rate and its clonogenicity in single-cell cultures compared to control cells and also to those transduced with the NBD mutant, whose behavior was generally more similar to that of the control cells. Conversely, *ZNF521* silencing led to an over 50% reduction of the clonogenic potential. Similar results were obtained when growth and clonogenicity were assessed in anchorage-independent culture conditions by measuring the potential of the cells to grow as spheroids and to form colonies in soft agar respectively.

The pro-clonogenic effects of ZNF521 in soft agar and in serum-free liquid cultures suggest that this factor may play a role in a stem-like population of cells in human medulloblastoma cell lines. However, unlike in primary tumors [47] the utility of stem cell markers in long-term human cell lines is not clear, and sphere counts cannot always be regarded as a faithful index of the numbers of immature cells within a culture [33-34]. It has been proposed that a correlation exists between the total cell expansion measured in the sphere assays and the rate of symmetric divisions in long-term proliferating cells [48]. In the light of this concept, the growth rate profiles obtained from the sphere assays (Fig. 2B) suggest that ZNF521 may enhance the self-renewal in a subset of primitive DAOY cells that could be related to medulloblastoma-initiating cells. This notion was supported by the increase of *in vivo* tumorigenicity we observed in DAOY cells expressing full-length ZNF521, as well as the decrease in development and growth rate of tumor xenografts following silencing of the abundantly-expressed *Zfp521* gene in cells from *Ptc1*^−/+^ medulloblastomas. In addition, ZNF521 enhanced the migratory ability of DAOY cells, which may contribute to the invasiveness of the tumorigenic cells.

Of particular interest is the finding that the functions of ZNF521 identified in DAOY cells seem dependent on the integrity of its NuRD-binding motif. We have determined, in proteomics analyses, that all known NuRD components are among the major interactors of ZNF521, and all NuRD subunits tested are found associated to ZNF521 in different cell types [4, 10; Bernaudo et al., unpublished data]. Despite this strong association, the NuRD-binding motif is not required for most of the biological effects of ZNF521/Zfp521 thus far discovered, aside from its partial contribution to the inhibition of the transcriptional activity of GATA1 [9] and EBF1 [10, 17].

The NuRD complex is composed by multiple subunits, whose assembly in variable combinations is believed to determine the specificity of the effects of the complex [49, 50]. NuRD recognizes a multitude of molecular partners, and displays a pleiotropic spectrum of transcriptional as well as non-transcriptional activities [51]. It participates in the control of several crucial processes including cell differentiation, cell cycle, apoptosis, genome stability, and tumor development - that it may promote or prevent depending on the molecular context.

The expression of both ZNF521 and all known NuRD members in medulloblastomas is at least equivalent to, and in some cases higher than, that of normal cerebellum (Suppl. Fig. 5); some of the NuRD components, in particular histone deacetylases, have been implicated in the control of regulatory pathways that govern the growth of MB cells [51-53] although it remains to be established whether these enzymes, that are also critical co-factors of other additional regulatory complexes [54], perform these functions in the context of the NuRD. Taken together, our data lend support to the idea that the interaction NuRD-ZNF521 may be relevant in the pathogenesis of human medulloblastoma, and warrant further studies aimed at confirming the importance of this interaction. In particular, it will be essential to determine the exact composition of NuRD complexes associated to ZNF521 in human MB cells and the biological effect of individual subunits and targets.

In conclusion, the data illustrated in this paper implicate the stem and progenitor cell factor ZNF521 and its association with the NuRD complex in the control of the growth of at least a subset of medulloblastomas. The role of ZNF521 in hematopoetic tissues and its effects on the clonogenic and *in vivo* growth of human and murine medulloblastoma cells suggests a potential requirement in stem-like tumor cells. However, better differentiated cells may also require its activity, and additional studies will be required to determine the precise contribution of ZNF521 to medulloblastoma pathogenesis and its potential utility as a candidate target for advanced therapeutic approaches in MB-initiating cells.

## METHODS

### Cell lines and culture conditions

DAOY, UW228, D283, HEK293T and NIH3T3 cells were cultured in Dulbecco's modified Eagle medium and the IM-9, THP1 and K562 in RPMI 1640, supplemented with 10% foetal bovine serum, 50U of penicillin and 50μg of streptomycin/ml, at 37°C in 5% CO_2_. All tissue culture reagents were from Life Technologies (Monza, Italy). DAOY and UW228 cells were validated at the Johns Hopkins Genetic resources core facility by PCR amplification of eight short tandem repeat (STR) loci plus a gender determining marker, Amelogenin. The PCR product was electrophoresed on an ABI Prism® 3730xl Genetic Analyzer using an Internal Lane Standard 600 (Promega). Data were analyzed using GeneMapper® v 4.0 software (Applied Biosystems).

### Expression vectors

The ZNF521 expression plasmids p3xFlagCMV7.1-ZNF521 and ZNF521 ΔNBD (lacking the first 13 amino acids that constitute the NuRD-binding motif) have been previously described (1, 4). The lentiviral vectors FUIGW-ZNF521 and FG12-H11 were previously described (23). To these, another shRNA vector (LV-H85) was added that carried the sequence:GCCCUCACUCUAUAACCUAAA derived from a Mission shRNA lentiviral vector (Sigma-Aldrich, Milan, Italy). For Zfp521 silencing, two Mission lenti-vectors were found effective and used in the relevant experiments as indicated:

LVp15: CGGCCCAUAUAUAUAUUUGUA;

LVp19: CCCUCAGUGUAACAAAGAAUU.

As a control, the non-target Mission lentiviral vector was used.

### Gene expression profiling of ZNF521 and of NuRD complex components in human medulloblastomas

Expression of ZNF521 mRNA and of the transcripts coding for all the known NuRD subunits was analyzed in a series of 436 medulloblastomas and 18 normal cerebellum profiles that were all profiled using the Affymetrix GeneChip® Human Genome U133 Plus 2.0 arrays. Data were combined from published series (24-27) and additional cases profiled either in Cambridge, UK (Mccabe et al., unpublished) or in Heidelberg, Germany (Kool et al., unpublished). All data were normalized using the MAS5.0 algorithm of the GCOS program (Affymetrix Inc, Santa Clara, CA, USA) and analyzed using the R2 software for analysis and visualization of microarray data (http://r2.amc.nl).

### Quantitative RT-PCR

cDNA was synthesized from 1μg of total cellular RNA isolated with Trizol (Life Technologies) and pre-treated with RNAse-free DNAseI (Promega, Milano, Italy) with SuperScript III reverse transcriptase and 2.5μM random hexamers (Invitrogen). Q-PCR reactions were carried out with the iQ SYBR green super mix (Bio-Rad Laboratories, Milano, Italy) in triplicate according to the manufacturer's instructions and analyzed using iQ5 multicolor detection system (Bio-Rad). One cycle of 3 min at 95°C for activation was followed by 45 cycles of 10 seconds at 95°C, 10 seconds at 60°C and 20 seconds at 72°C and then a melting curve. Relative gene expression was determined using the comparative threshold cycles Ct method, normalizing for endogenous GAPDH and expression ratio was calculated as 2^−ddCt^. The specificity of amplification was confirmed by the dissociation curves of the amplicons. All data are mean values +/− standard deviations from at least three independent experiments using different RNA preparations.

Primers used for q-PCR were the following for human mRNA transcripts.

ACCATCTTCCAGGAGCGAG GAPDH-FWD

TCACGCCACAGTTTCCCGGA GAPDH-REV

CCACATCCAAACCATCCACCG ZNF521-FWD

CAGGTGGCACTGGAGTTTGGC ZNF521-REV

### Antibodies

A rabbit antibody (I88) against an internal ZNF521 peptide (VEAAPPIPKSRGRKR) was produced (Primm, Milano, Italy) and affinity-purified. In non-reducing conditions, this antibody predominantly recognizes a dimeric form of ZNF521 at 300 kDa in western blotting at a dilution 1:1000. Rabbit anti-EHZF (S15), raised against a peptide encompassing the same region as that used to produce the I88 Ab, was from Santa Cruz Biotechnology (Heidelberg, Germany) and was used at 1:1000.

### Western blotting

Nuclear extracts prepared in 400mM NaCl, 20mM HEPES pH 7.9 in the presence of protease inhibitors and in the absence of EDTA/DTT, were electrophoresed on NuPAGE Novex Bis-Tris 4-12% gels under non reducing conditions, electro blotted onto nitrocellulose membranes, stained with Ponceau S and blocked with 5% blocking reagent (BioRad). The antigens were detected using primary antibody [I88 (Primm) or anti-EHZF S15 (Santa Cruz) (1:1000) or anti-HDAC1 (Sigma Aldrich, Milano, Italy)(1:10000)], secondary HRP-conjugated anti-rabbit, and Chemi-luminescence Luminol Reagent (Santa Cruz). In Fig. 1B total cell extracts were analyzed; here GAPDH, used as an internal control in lieu of HDAC1, was detected using a mouse anti-GAPDH mAb (Santa Cruz, sc-166574) and HRP-rabbit anti-mouse.

### Lentiviral vector production

The production of the lentiviral vectors described in this paper was performed as previously detailed [10, 55, 56].

### Tumor Xenografts in Nude Mice

Five-seven week-old female athymic nude mice were purchased from Harlan Italy and housed under pathogen-free conditions throughout the experiments. DAOY transduced with the relevant lentiviral vectors were resuspended at the concentrations desired into single-cell suspensions in 200 μL of PBS 1x and injected subcutaneously in the back of the mice. Tumor growth was monitored biweekly over at least 4 months. The dates at which a palpable tumor first arose were recorded. Once the tumor size reached a volume ≥ 300 mm^3^, the mice were sacrificed and the tumors weights recorded. For the histological and immunohistochemical analyses, tumor masses were sliced on a cryostat at 6.0 μm thickness, stained with hematoxylin/eosin stain or with a rabbit anti-ZNF521 antibody (Sigma HPA023056) followed by AlexaFluor 568-conjugated goat anti-rabbit IgG (Life Technologies). Images were captured with a Leica TSC SP2 confocal microscope at 20x magnification, and acquired using Leica Confocal Version 2.6.1.

### Lentiviral transduction of mouse MB cells and *in vivo* tumorigenicity studies

Medulloblastoma tumors from cerebella of postnatally irradiated Ptch^−/+^ mice were serially passaged *in vivo* subcutaneously. When tumors reached a volume ranging between 400 and 1000 mm^3^, mice were sacrificed and tumors explanted to obtain single cell suspensions by mechanical and enzymatic dissociation with Collagenase XI and Hyaluronidase (Sigma). Single cells were resuspended in NPBM (Neural Progenitor Basal Medium) supplemented with EGF, FGF, NSF-1 and GA (Lonza, Basel, Switzerland) and maintained in culture for 48 hours, the time necessary for transduction and, when indicated, for 96 hours for proliferation assays. Viable cells were counted by using a Guava instrument and 400.000 cells per well were transduced with 1 ml of the preparation of recombinant lentivirus in the presence of 8 μg/ml of Polybrene. Transduction efficiency was monitored by flow cytometry 48 hours post infection. For the *in vivo* tumorigenicity assays, immediately after the FACS analysis 50.000 transduced medulloblastoma cell suspensions/ mouse were injected subcutaneously in the presence of 50% Matrigel (BD Biosciences, Buccinasco, Italy) in 5-weeks old CD1 nude mice. Tumor volumes were measured twice a week with a Digimatic Caliper (Mitutoyo, Milano, Italy); body weight and clinical signs were monitored twice a week.

Proliferation of transduced single cell suspensions was determined by a Brdu incorporation assay (Roche, Milano, Italy) according to manufacturer's instructions; light emission was measured in a microplate luminometer (Top Count).

All procedures involving animals and their care conformed to institutional guidelines that comply with national and international laws and policies. The *in vivo* tumor growth experiments were conducted according to the published guidelines for the welfare and use of animals in cancer research. All animals were examined daily for a decrease in physical activity or other signs of disease; severely ill animals were euthanized by CO_2_ asphyxiation.

### Cell growth assays

Cell proliferation was measured by MTS (3-(4,5-dimethylthiazol-2-yl)-5-(3-carboxymethoxy-phenyl)-2-(4-sulfophenyl)-2H-tetrazolium) colorimetric assay. Cells were seeded at 5×10^3^ per well in in 96-well plates in 10% FBS-containing medium and assayed after 24, 48 and 72 hours of incubation. At each time point, 20 μl of MTS solution was added to the well. After 1h incubation at 37°C the absorbance, which is proportional to the number of viable cells, was measured at λ = 490 nm. Experiments were performed in triplicate.

Adherent cell growth was also monitored by plating 5 × 10^4^ transduced cells per 100 mm tissue culture treated dish in 10 ml DMEM 10% FBS. At 48h intervals the cells were trypsinized, counted and replated at 5×10^4^/dish. The average of three independent cell counts, normalized to account for the dilutions performed in the individual cultures at each replating, was plotted against time to obtain a cumulative growth curve for each cell population.

### Tumor sphere culture assay

For this assay transduced DAOY cells were trypsinized, washed in Neurobasal medium A (Life Technologies) and resuspended at 5×10^5^ cells/10mm non-adherent Petri dishes in 7 ml of the same medium containing 2 mM L-glutamine, N2 supplement, B27 supplement, 20 ng/mL hrEGF (PeproTech, London, UK), 20 ng/mL hrbFGF (PeproTech) and 50 μg/mL BSA (Life Technologies). Fresh growth factors were added to the cells twice a week. Neurospheres were disaggregated with trypsin in single-cell suspensions after 7 and 14 days and reseeded at 5 ×10^5^ per dish in fresh medium to form secondary and tertiary spheres. Cumulative total numbers of cells from the spheroid cultures were calculated.

### Single-cell clonogenic assay

Transduced cells were grown to 60% confluency, trypsinized and an average of 0.3 cells/well were seeded in 96-well plates in DMEM 10% FBS. After 14 days, colonies containing >100 cells were scored at 10x magnification.

### Soft agar colony formation assay

The base layer for this assay consisted of serum-supplemented medium containing 1% agar (18300012, Life Technologies). The cell layer contained 1×10^4^ cells per 35mm dish, admixed with serum-supplemented medium in 0.5% agar on top of the base layer. After 3–4 weeks, colonies larger than 50 μM in four high-powered fields per well were scored by computer-assisted image analysis with the MCID Elite software. Each experiment was performed at least twice in triplicate.

### Wound healing assay

Transduced cells were cultured until >90% confluence in 35mm dishes. After 24 hours the cell monolayer was scratched using a sterile 200 μl pipet tip, washed with PBS and incubated for up to 24 h in DMEM 10% FBS. Images were captured by phase contrast microscopy at 10x magnification, at the beginning and at regular intervals during cell migration. The experiment illustrated in Suppl. Fig. 1 was performed in the presence of 5μg/ml Mitomycin C.

### Statistical analyses

P-values were obtained by applying a one-tailed, type 2 t-test (assuming equal variances) using Microsoft Excel and GraphPad Prism 4.0b. Data illustrated in Fig. 5D were analyzed using Fisher's exact test.

## Supplementary Figures


