# Supplementary material for: Critical role of zinc finger protein 521 in the control of growth, clonogenicity and tumorigenic potential of medulloblastoma cells
Source: Oncotarget. 2013 Jul 27;4(8):1280–92. doi: 10.18632/oncotarget.1176 (PMC3787157; doi:10.18632/oncotarget.1176)
Supplement: Supplementary file 1 [file oncotarget-04-1280-s001.pdf]

Critical role of zinc finger protein 521 in the control of growth, clonogenicity and tumorigenic potential of medulloblastoma cells - Spina et al

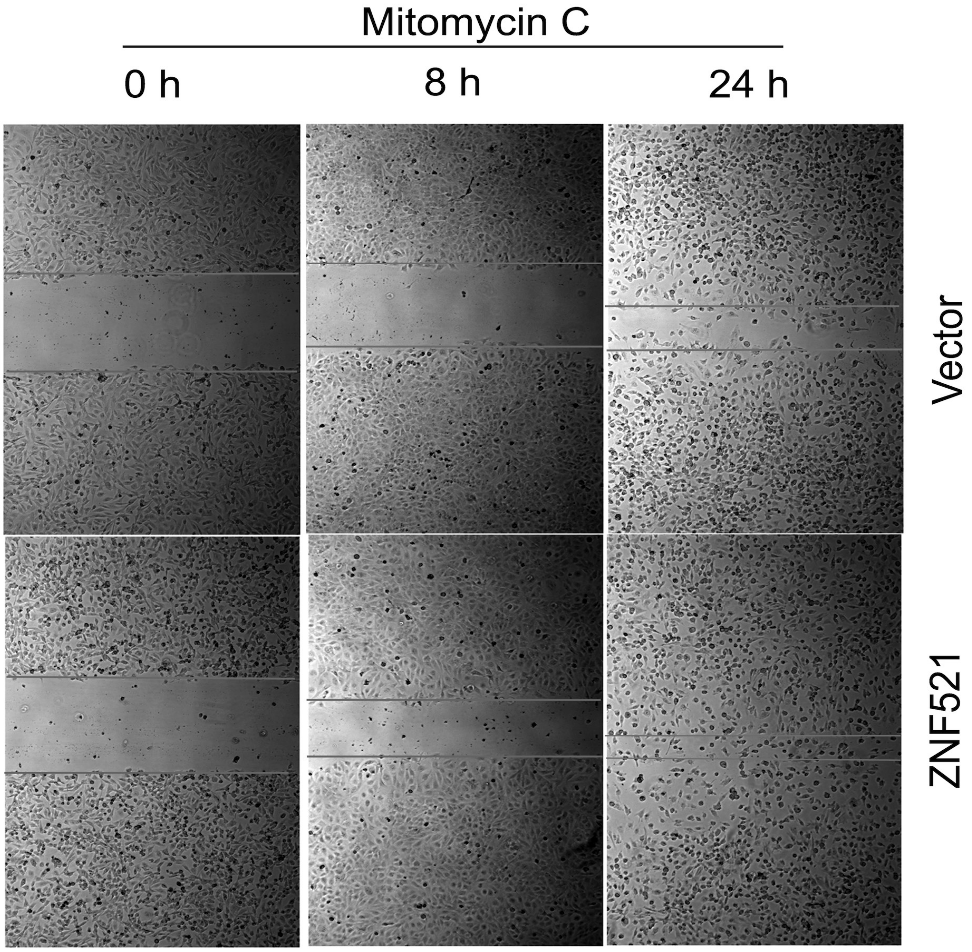

Suppl Fig. 1: Wound-healing assay in the presence of Mitomycin C. The assay was performed as indicated in materials and methods. This figure illustrates a representative experiment of a set of 3.

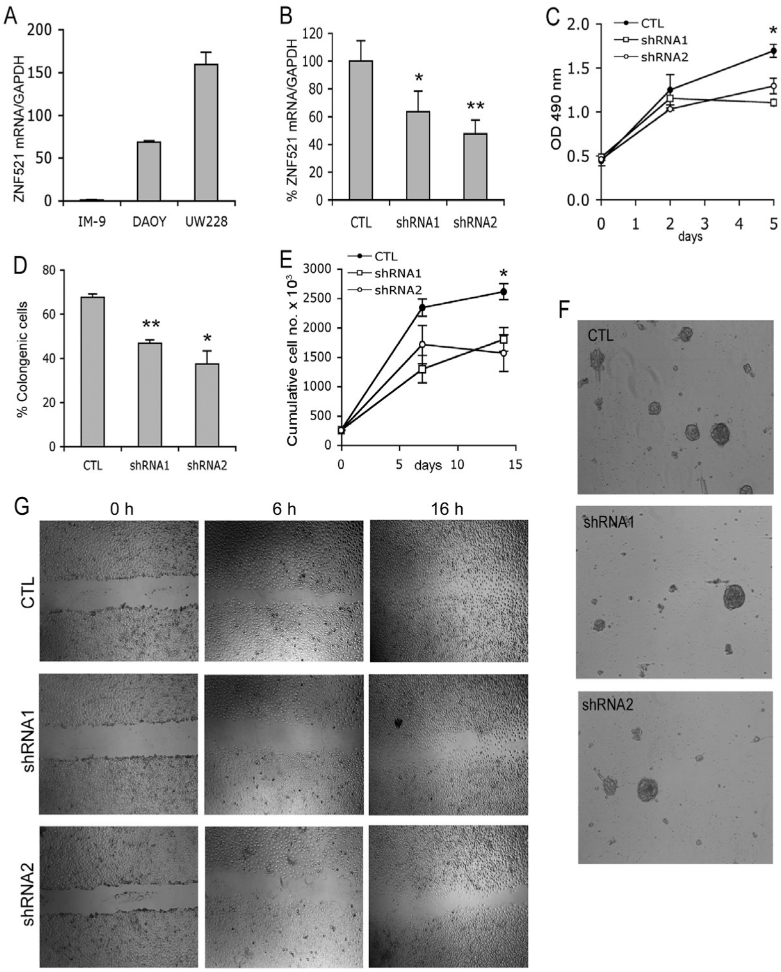

Suppl. Fig. 2: Silencing of ZNF521 impairs the growth, clonogenicity, sphere formation and migration of UW228 human medulloblastoma cells. Panel A: ZNF521 expression in UW228 was measured by Q-RT-PCR as described in materials and methods in comparison with DAOY and IM-9 cells. Panel B: Modulation of ZNF521 expression in UW228 cells transduced with the vector FG12 (CTL) and the shRNA-containing FG12-H11 (shRNA1) and LV-H85 (shRNA2), measured by Q-RT-PCR. Panel C: the growth of UW228 transduced as described above was measured by MTS assay. Solid circles: CTL; open squares: shRNA1; open circles: shRNA2. Panel D: clonogenicity of DAOY cells transduced with FG12, FG12-H11 (shRNA1) or LV-H85 (shRNA2) in single-cell culture conditions. Panel E: growth of UW228 transduced as described above in anchorage-independent conditions. The cultures were performed as described in materials and methods. Solid circles: CTL; open squares: shRNA1; open circles: shRNA2.Representative spheres are shown in Panel F. Panel G: Wound-healing assay of UW228 cells transduced with the vector FG12 (CTL) and the shRNA-containing FG12-H11 (shRNA1) and LV-H85 (shRNA2). The assays were performed as described in materials and methods. The results shown in the figure illustrate representative experiments of a set of 3.

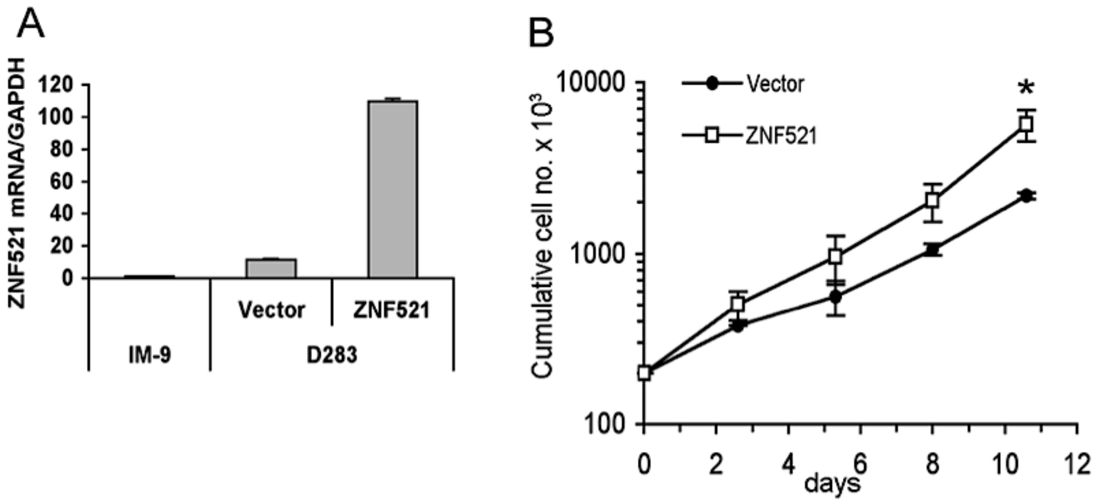

Suppl. Fig. 3. Enforced expression of ZNF521 enhances the growth of D283 human medulloblastoma cells. Panel A: ZNF521 expression in D283 cells transduced with FUIGW (Vector) or FUIGW-ZNF521 (ZNF521) was measured by Q-RT-PCR as described in materials and methods in comparison with IM-9 cells. Panel B: the growth of D283 cells transduced as described above was measured by cell counts at the indicated times, as described in materials and methods.

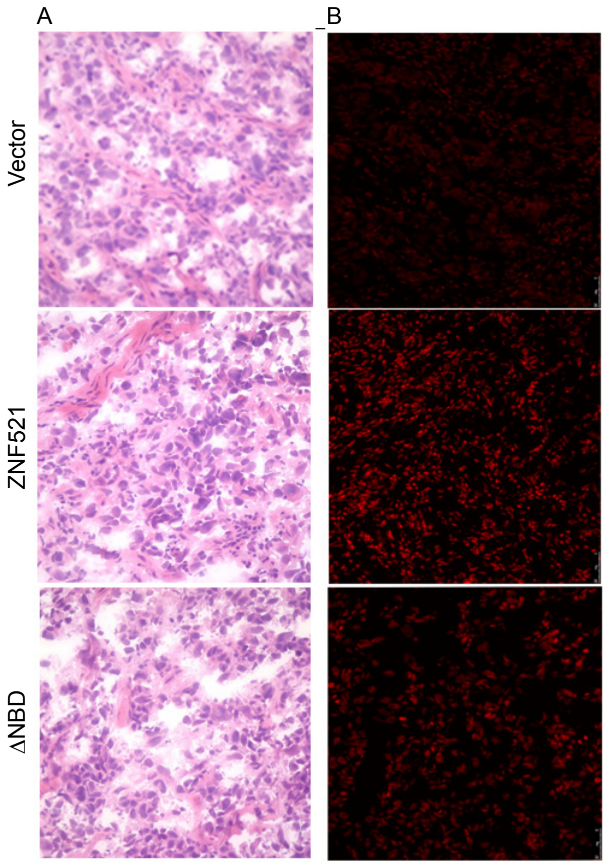

Suppl. Fig. 4. Histology and ZNF521 expression of DAOY-derived tumors in nude mice. Panel A: Tumors from mice of exp. 2 stained with hematoxylin/eosin. Panel B: The detection of ZNF521 protein was carried out as detailed in materials and methods by indirect immuno-fluorescence.

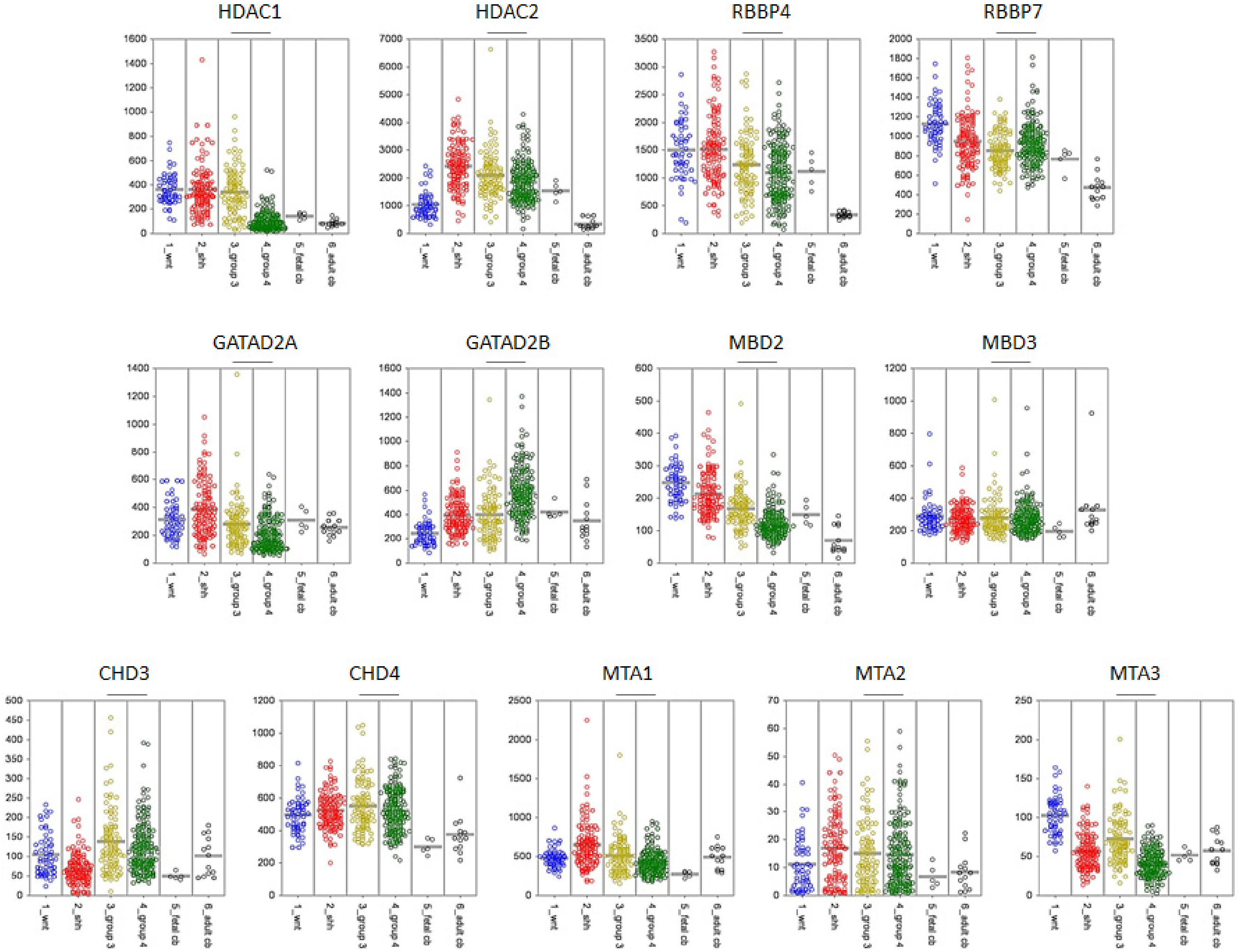

Suppl. Fig. 5. Expression of NuRD complex members in human medulloblastomas. The comparative analysis of 436 medulloblastomas and 18 samples of normal fetal or adult cerebellum was carried out as described in materials and methods.
